# Dimethyloxalylglycine pretreatment of living donor alleviates both donor and graft liver ischemia-reperfusion injury in rats

**DOI:** 10.3389/fphar.2023.1341575

**Published:** 2024-01-09

**Authors:** Degong Jia, Minjie Zhao, Jiefu Luo, Shengwei Li, Jianping Gong, Mingxiang Cheng

**Affiliations:** Department of Hepatobiliary Surgery, The Second Affiliated Hospital of Chongqing Medical University, Chongqing, China

**Keywords:** living donor liver transplantation, hypoxia-induced transcription factor 1, ischemia-reperfusion injury, dimethyloxalylglycine, oxidative stress

## Abstract

**Background:** Under the circumstance of the increasing waiting list for liver transplantation, living donor liver transplantation (LDLT) can alleviate the shortage of liver donors to some extent. However, how to reduce both donor and graft ischemia-reperfusion injury (IRI) is still an unsolved problem in LDLT. Hypoxia-induced transcription factor 1 (HIF1) activation is considered an important mechanism of cellular adaptation to hypoxia, and early activation of HIF1 may be a new way to alleviate liver IRI. Therefore, we aimed to investigate the impact of the HIF1 stabilizer dimethyloxalylglycine (DMOG) on IRI and the survival rate of donors and recipients of rat LDLT.

**Methods:** Seventy percent partial liver resection and 30% partial liver transplantation were used to simulate donor and recipient of clinical LDLT. Rats were treated with DMOG (40 mg/kg) or with an equivalent amount of saline. The expression of HIF1 and downstream targets was analyzed after 2 h of reperfusion. Liver function and histopathology, apoptosis and oxidative stress levels were detected 6 h after reperfusion. At the same time, the 7-day survival rate of rats was calculated.

**Results:** DMOG pretreatment significantly reduced IR-induced injury in the donor and recipient, which was manifested by reducing liver function damage and promoting tissue recovery. Meanwhile, compared with the untreated group, the oxidative stress level and the cell apoptosis rate were decreased in the group pretreated with DMOG. In addition, the transcription and expression of HIF1 target genes in the DMOG group were significantly enhanced. Remarkably, DMOG also increased the survival rate of the recipient.

**Conclusion:** This study provides the first evidence that DMOG pretreatment of donors significantly alleviates liver IRI in both donors and recipients and increases the survival rate of recipients in LDLT. Therefore, DMOG may be a promising strategy for improving LDLT in the future.

## Graphical Abstract

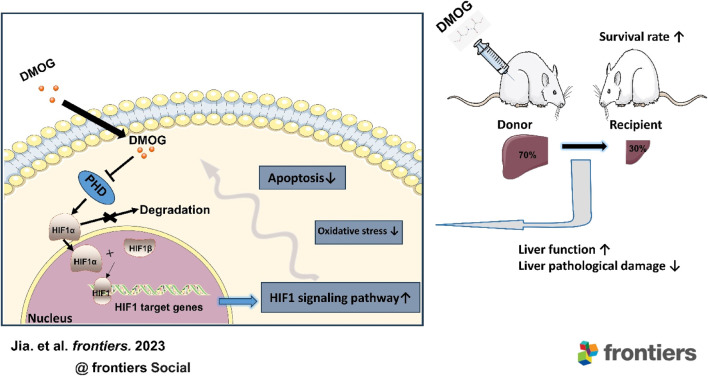

## 1 Introduction

Liver transplantation (LT) is the best treatment option for end-stage liver disease, but it has been limited by the shortage of donor organs. Since the first living donor liver transplantation (LDLT) was carried out by Strong et al., in 1989, LDLT has greatly expanded the donor pool (Strong et al., 1990). In Asia, the low organ donation rate due to legal, religious and cultural restrictions is particularly severe, and the need for donors can only be met through large-scale LDLT. Therefore, LDLT flourishes in Asia accounted for 90% of LT activity (Chen et al., 2013). Nevertheless, the safety of LDLT donors, especially in adults, has limited the development of LDLT to some extent (Park et al., 2019). Both donor liver and graft have to undergo ischemia and hypoxia, leading to ischemia-reperfusion injury (IRI), which seriously affects the clinical prognosis of LDLT. It is a priority to find an intervention that improves both donor and graft outcomes. However, to date, few studies have focused on mitigating IRI in both donors and recipients at the same time, and there are no satisfactory ways to improve the prognosis of LDLT.

The intracellular response to oxygen tension is well known to be regulated by hypoxia-inducible factors (HIFs). HIF1 is a heterodimer consisting of a constitutive β subunit and an oxygen-dependent α-subunit (HIF-1α). Under normal oxygen conditions, HIF-1α is rapidly degraded by O_2_-dependent prolyl hydroxylase domains (PHDs) to ensure that HIF-1α levels remain low. When cells are hypoxic, PHD activity is reduced, and HIF-1α accumulates and binds to HIF-1β to form the active transcription factor HIF-1. HIF1 then binds to hypoxia response elements in the promoter-enhancer region of the target gene, thereby regulating more than 100 target genes to regulate adaptation to hypoxia (Eckle et al., 2008). Stabilization of HIF-1α can enhance the transcription and expression of downstream essential target genes under hypoxic conditions and alleviate IRI (Harnoss et al., 2022; Yang et al., 2023).

Dimethyloxalylglycine (DMOG) is a permeable analog of oxyglutaric acid that inhibits HIF-1α degradation and causing its accumulation in the nucleus by inhibiting PHD activity, thereby significantly inducing downstream gene expression of HIF1. Pretreatment with DMOG appears to improve the early recovery of graft function after heart transplantation (Hegedus et al., 2016). However, it is unclear whether DMOG therapy can alleviate donor and recipient IRI in LDLT.

Therefore, in this study, 70% hepatectomy and 30% partial LT were used to observe the effects of DMOG on donor and recipient IRI in rat LDLT.

## 2 Materials and methods

### 2.1 Animals

Male SD rats (aged 7–8 weeks, weighing 250–280 g, Hunan SJA Laboratory Animal Co., Ltd., Hunan, China) were used in this study and raised in a specific pathogen-free room at 22°C ± 2°C with a 12 h:12 h light and dark cycle, allowing free access to drinking water and standard food. All animal experiments were conducted according to protocols approved by the Ethics Committee of Chongqing Medical University (Chongqing, China) (Approval NO. IACUC-SAHCQMU-2023-0018). The animal procedures were carried out in accordance with the recommendations provided in the Guide for the Care and Use of Laboratory Animals (National Institutes of Health, Bethesda, MD, United States).

### 2.2 Materials and reagents

DMOG (No. A4506) was purchased from APExBIO Technology LLC (Huston, United States). Specific antibodies for HIF-1α, vascular endothelial-derived growth factor (VEGF), phosphoinositide-dependent protein kinase 1 (PDK-1), inducible nitric oxide synthase (iNOS), glucose transporter 1 (Glut1), heme oxygenase-1 (HO-1), glyceraldehyde-3-phosphate dehydrogenase (GAPDH) and β-actin were purchased from Abcam (Cambridge, UK). The specific antibodies for erythropoietin (EPO) were purchased from Proteintech Group, Inc (Hubei, China). The assay kits for superoxide dismutase (SOD) and catalase (CAT) were purchased from Nanjing Jiancheng Bioengineering Institute (Jiangsu, China). The assay kits for malondialdehyde (MDA) and glutathione (GSH) were purchased from Servicebio (Hubei, China). The assay kits for alanine aminotransferase (ALT) and aspartate aminotransferase (AST) were purchased from Rayto Life Sciences Co., Ltd. (Shenzhen, China).

### 2.3 Experimental groups

**FIGURE 1:**
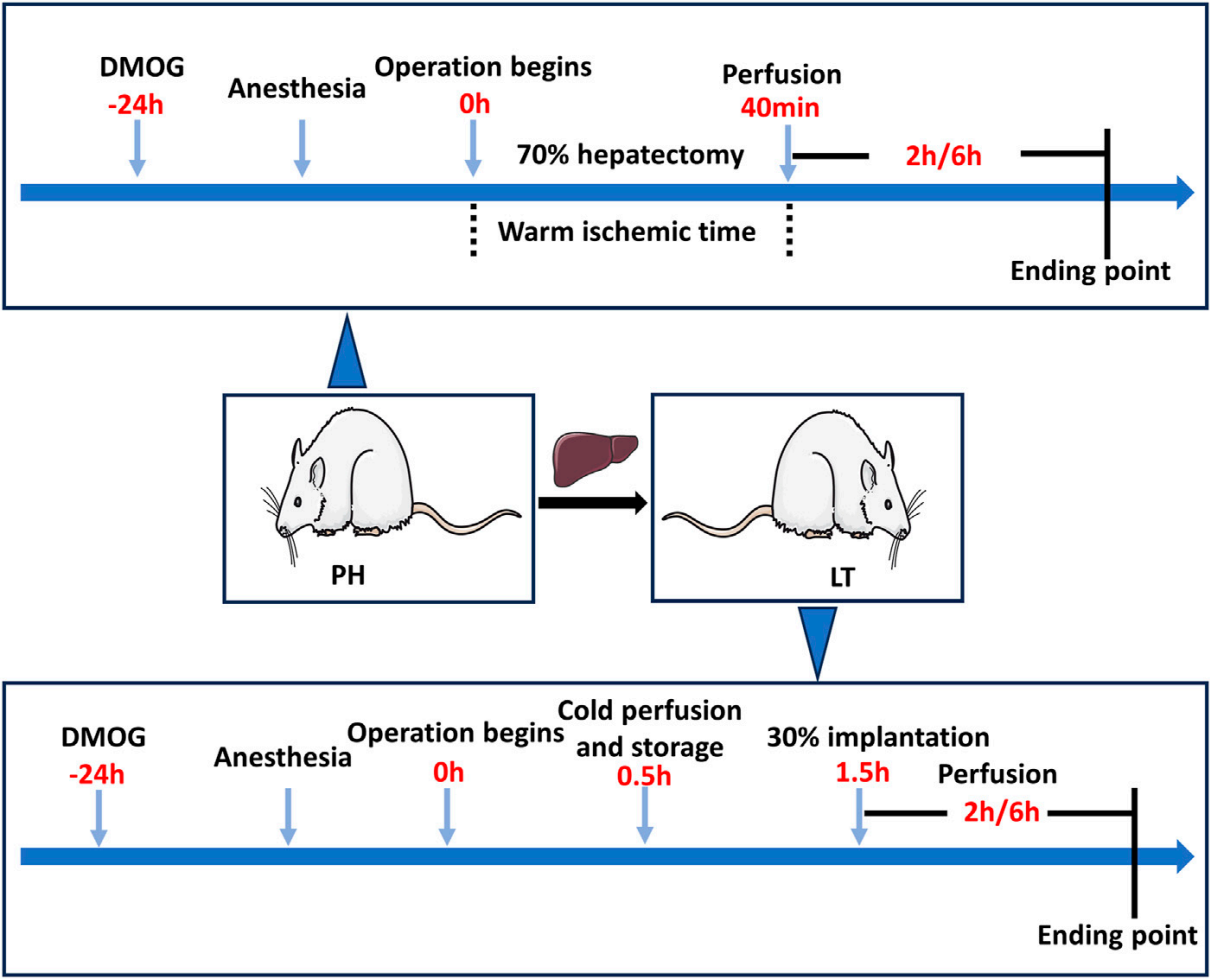
Diagram of the study protocol. In the PH + DMOG and LT + DMOG groups (treatment), 40 mg/kg DMOG was injected intraperitoneally for drug delivery to the donor livers. The rats in the sham, PH and LT groups were injected intraperitoneally with an equivalent amount of saline. In the Sham groups, only perihepatic ligaments were freed, and rats were subjected to 2 or 6 h of reperfusion without ischemia.

A 70% hepatectomy model was established to simulate the clinical conditions of LDLT donors, and a 30% partial LT model was established to simulate the clinical conditions of LDLT recipients. To minimize experimenter bias, the investigator performing the procedure is unaware of the treatment conditions. The experiment was divided into two parts, and Figure 1 shows a schematic diagram of the experimental protocol.

In experiment 1, the rats were randomly divided into the following 3 groups. 1) Sham 1 group: only perihepatic ligaments were freed, and 1 mL of saline was intraperitoneally injected 24 h before surgery. 2) Partial hepatectomy (PH) group: the rats underwent 70% hepatectomy and were injected with 1 mL saline 24 h before surgery. 3) In the PH + DMOG group, the rats were intraperitoneally injected with DMOG (40 mg/kg in 1 mL saline) 24 h before 70% hepatectomy.

In experiment 2, the rats were randomly divided into the following 3 groups. 1) Sham 2 group: only perihepatic ligaments were freed, and 1 mL saline was injected 24 h before surgery. 2) LT group: A 30% LT model was established, and the donor rats were injected with 1 mL saline. 3) LT + DMOG group, and the donor rats were injected (40 mg/kg in 1 mL saline) 24 h before surgery.

Animals were sacrificed at 2 and 6 h postreperfusion or followed for survival on day 7, and the serum and liver tissue were immediately collected for further analysis. The expression of HIF1 and downstream targets was analyzed after 2 h of reperfusion. Liver function and histopathology, apoptosis and oxidative stress levels were detected 6 h after reperfusion.

### 2.4 Surgical procedure

We established a 70% hepatectomy model with reference to Okumura et al., and all procedures were performed under inhalation anesthesia (Okumura et al., 2016). The abdomen was incised through the midline, and a noninvasive clamp was used to block the blood supply to the left and middle hepatic lobes. Subsequently, the left lobe, left portion of the medial lobe, right inferior and superior lobes, and caudate lobes were immediately ligated and excised, and only the right portion of the medial lobe was left. After 40 min of ischemia, the clamp was removed. The abdomen was sutured, and the rats were awakened.

The model of 30% LT in rats was modified on the basis of a previously described protocol (Liang et al., 2021). At the same time, referring to our previous methods, cuff techniques were used for anastomosis of the subhepatic inferior vena cava (IVC) and portal vein, and magnetic rings were used for anastomosis of the suprahepatic IVC (Pan et al., 2018). The brief steps are as follows.

Donor liver procurement: Biliary duct intubation was performed after the perihepatic ligaments were freed and relevant vessels were exposed. The abdominal aorta was perfused, and then the donor liver was obtained and stored in lactated Ringer’s solution at 0–4°C.

Graft preparation: The left and medium lobes were ligated and removed. The reduced graft accounted for approximately 30% of the donor liver mass. Subsequently, the portal vein and subhepatic IVC were cannulated with cuffs, and a magnetic ring was prepared for the suprahepatic IVC.

Recipient operation: After laparotomy, the perihepatic ligaments and bile duct were set free, the hepatic artery and adrenal vein were ligated, the portal vein and subhepatic IVC were clamped, and compound sodium chloride solution was used to expel blood from the liver. Subsequently, the recipient liver was removed after the suprahepatic IVC was clamped. The suprahepatic IVC was everted through the magnetic ring, and the graft was placed in place. The anastomosis of the recipient’s and graft’s suprahepatic IVCs was completed through magnetic attraction. The anhepatic stage was completed after the portal vein and subhepatic IVC cannula. In our experiment, the anhepatic phase was adjusted to exactly 13 min. The abdominal cavity was rinsed with warm saline, and the intestinal tube was reset. The abdomen was stratified closed after the graft returned to a rosy color and bile began to be secreted.

### 2.5 Liver function

ALT and AST levels in serum were detected by an automatic biochemical analyzer (Rayto, China) as standard indexes of hepatocyte injury.

### 2.6 Histopathological analysis of rat liver

The liver tissue was fixed in 10% paraformaldehyde for 24 h, embedded in conventional paraffin, and sliced continuously with a length of 4 μM. Hematoxylin and eosin (HE) staining was used to observe histological changes under light microscopy, and quantification was performed by two pathologists based on Suzuki’s score (necrosis, sinusoidal coagulation, and ballooning generation).

### 2.7 *In situ* apoptosis detection

Apoptosis was detected *in situ* by terminal-deoxynucleotidyl transferase-mediated nick end labeling (TUNEL). Four high-power fields (magnification, 400) were randomly selected per field, and Halo (version 3.0.311.314) software was used to quantify the number of positive and total cells in each field. The percentage of TUNEL-positive hepatocytes was calculated as the number of TUNEL-positive cells divided by the total number of cells.

### 2.8 Oxidative stress levels measure

One hundred milligrams of fresh liver tissue was weighed and added to 9 volumes of physiological saline to produce a 10% tissue homogenate. Subsequently, the homogenate was centrifuged at 3,000 r/min, and the supernatant was taken to determine the contents of MDA, SOD, GSH, and CAT.

### 2.9 PCR

Total RNA was extracted using a SteadyPure universal RNA Extraction Kit II (Accurate Biology, AG21022, China) according to the manufacturer’s instructions. RNA was reverse transcribed using the Evo M-MLV Mix Kit with gDNA Clean for qPCR (Accurate Biology, AG11728, China). The acquired cDNA was used to perform quantitative real-time PCR by using the SYBR Green Premix *Pro Taq* HS qPCR Kit (Accurate Biology, AG11701, China). The following primers were used to amplify the mRNA: HIF-1α (Fw: 5′-TAG​GGA​TGC​AGC​ACG​ATC​TC-3′; Rev: 5′- GTG​GCA​ACT​GAT​GAG​CAA​GC-3′), VEGF (Fw: 5′-AGG​GTC​AAA​AAC​GAA​AGC​GC-3′; Rev: 5′- CGC​GAG​TCT​GTG​TTT​TTG​CA-3′), iNOS (Fw: 5′-CTT​GGA​GCG​AGT​TGT​GGA​TTG-3′; Rev: 5′- CCT​CTT​GTC​TTT​GAC​CCA​GTA​GC-3′), PDK-1 (Fw: 5′-CCA​TAT​CAC​GCC​TCT​ATG​CAC-3′; Rev: 5′-TCT​TTC​GAT​GGA​CTC​CGT​TG-3′), EPO (Fw: 5′-ATT​CCT​CCC​AGC​CAC​CAG​A-3′; Rev: 5′- ACC​CGA​AGC​AGT​GAA​GTG​AG-3′), Glut1 (Fw: 5′-CTC​GGG​TAT​CGT​CAA​CAC​GG-3′; Rev: 5′-CCA​GCC​AGA​CCA​ATG​AGG​TG-3′) and HO-1 (Fw: 5′-GTG​ACA​GAA​GAG​GCT​AAG​AC-3′; Rev: 5′- GTA​GTA​TCT​TGA​ACC​AGG​CTA​G -3′). To normalize the cycle threshold to an endogenous control, the following primers were used: 5′-GGC​ACA​GTC​AAG​GCT​GAG​AAT​G- 3′(forward) and 5′-ATG​GTG​GTG​AAG​ACG​CCA​GTA -3′ (reverse) for rat GAPDH mRNA. The relative gene expression was determined by calculating the expression ratio of the gene of interest to GAPDH.

### 2.10 Western blot

Protein was obtained from liver tissue lysates, and protein from each sample was separated using sodium dodecyl sulfate‒polyacrylamide gel electrophoresis and transferred to polyvinylidene fluoride membranes, which were then blocked with 5% nonfat milk in TBST. Membranes were incubated with the respective primary antibodies at 4°C overnight. The corresponding secondary antibodies were incubated for 2 h at room temperature. Blots were detected and visualized using Image Lab software (version 6.1).

### 2.11 Statistical methods

GraphPad Prism software (version 8.0.2) was used, and the results are expressed as the mean ± SEM. One-way analysis of variance was used for comparison among the three groups, and Tukey’s *post hoc* analysis was performed to compensate for multiple comparisons. The survival rate was calculated by the Kaplan-Meier method. *p* < 0.05 was considered statistically significant.

## 3 Results

### 3.1 DMOG pretreatment alleviated hepatic IRI in PH and partial LT rats

**FIGURE 2:**
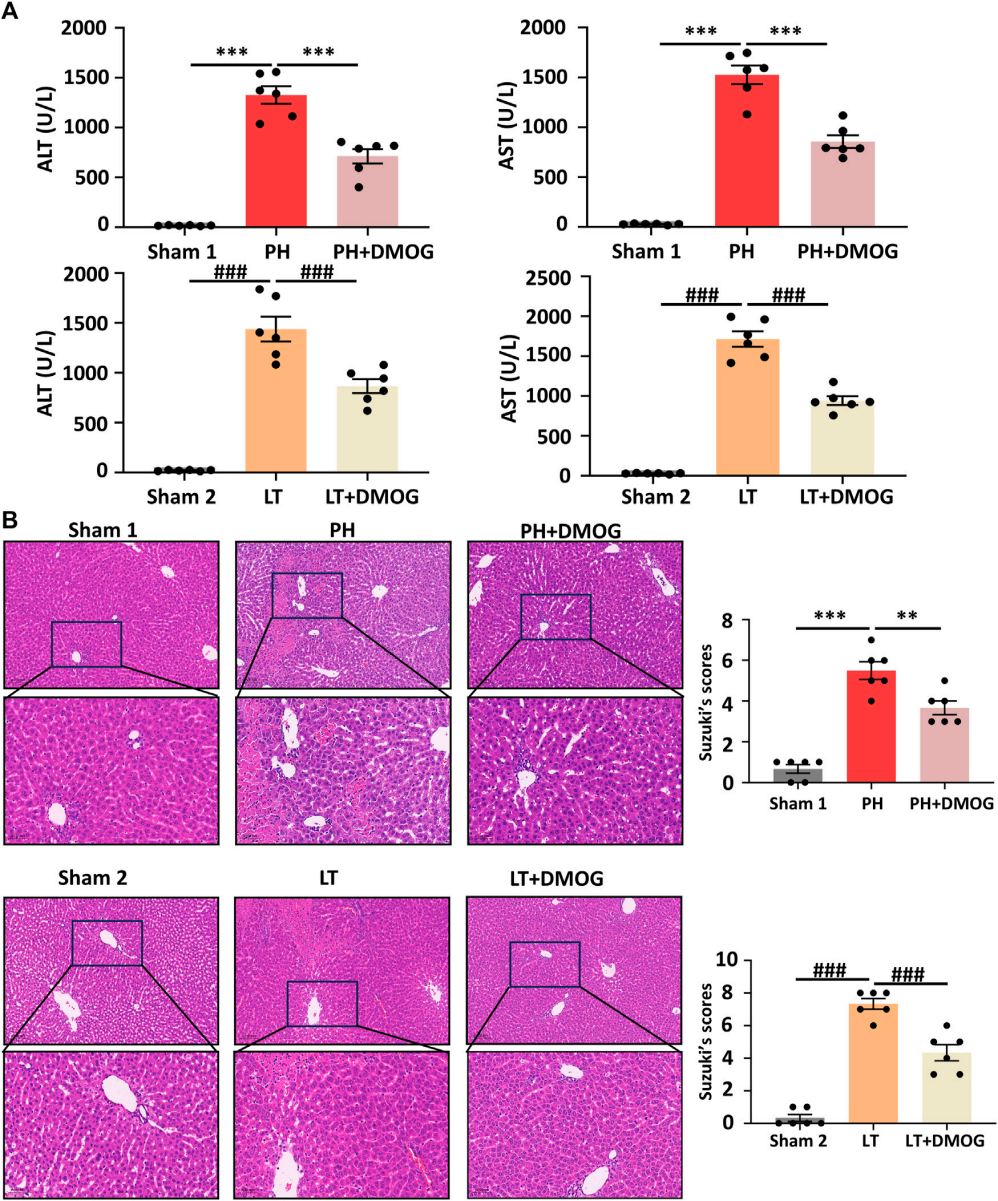
DMOG pretreatment alleviated hepatic IRI in PH and partial LT rats. ALT and AST assay **(A)**. HE staining. Scale bar: upper: 100 μm, lower: 50 μm **(B)**. *p < 0.05; **p < 0.01; ***p < 0.001. ^#^ *p* < 0.05; ^##^ *p* < 0.01; ^###^ *p* < 0.001. DMOG, dimethyloxalylglycine; IRI, ischemia–reperfusion injury; LT, liver transplantation; PH, partial hepatectomy.

We evaluated the effect of DMOG on liver function and morphological impairment 6 h after reperfusion. First, liver function was assessed by ALT and AST. As shown in Figure 2A, compared with the PH group, DMOG treatment significantly reduced ALT and AST levels. Compared with the LT group, DMOG treatment also reduced ALT and AST levels to some extent. Subsequently, we assessed liver morphological damage by HE staining. Six hours after reperfusion, the livers of rats in the PH and LT groups showed severe pathological changes, including severe lobular distortion of the liver, partial necrosis, hepatic sinus congestion, ballooning, and periportal edema. In contrast, DMOG pretreatment significantly mitigated these histological changes, manifested by preservation of lobular architecture and reduced congestion and necrosis. There was no doubt that the Suzuki’s score in the DMOG pretreatment group was significantly lower than that in the PH and LT groups. Representative histological sections are depicted in Figure 2B.

### 3.2 DMOG pretreatment attenuated cell apoptosis in PH and partial LT rats

**FIGURE 3:**
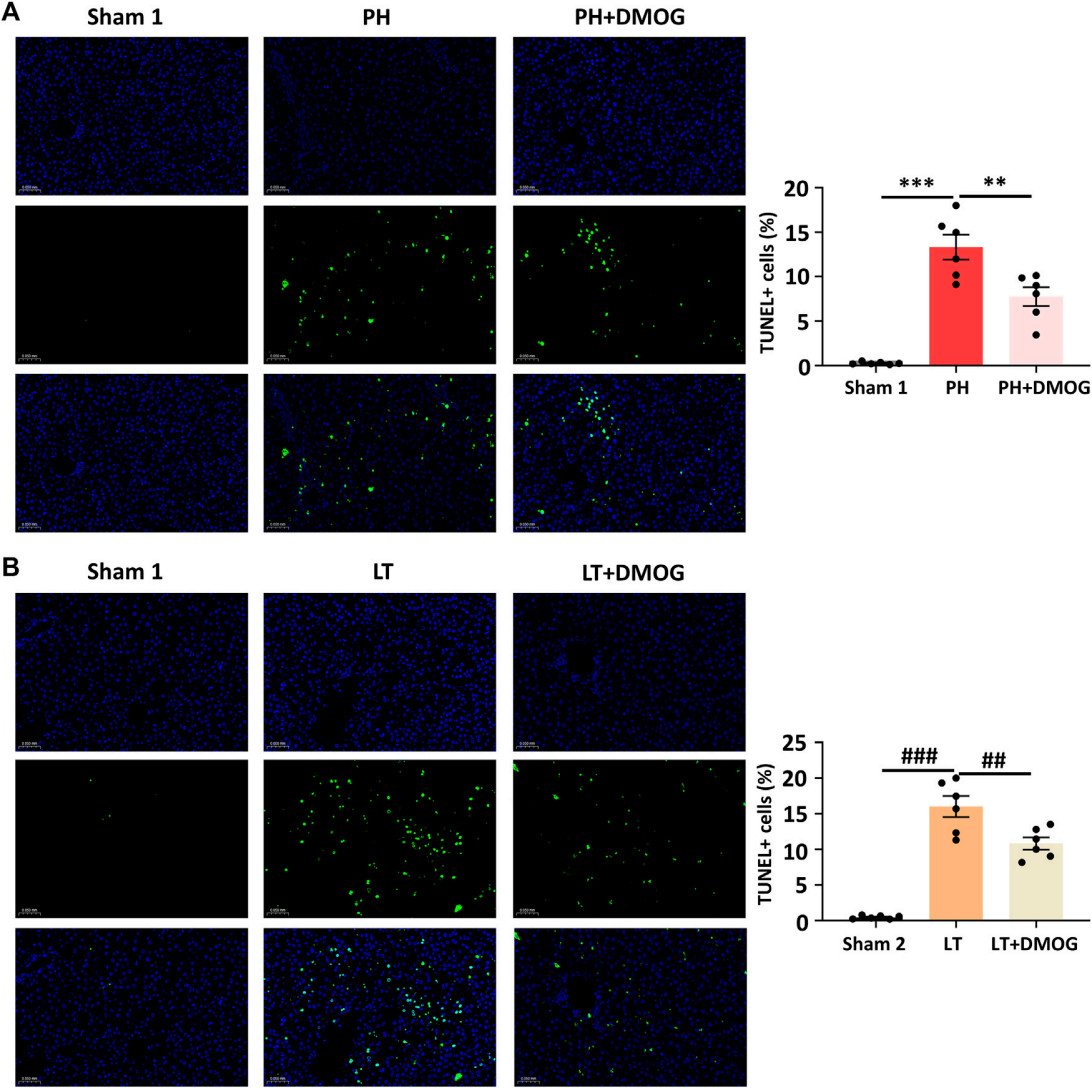
DMOG pretreatment attenuated cell apoptosis in PH and partial LT rats. TUNEL assay of PH. Scale bar: 50 μm **(A)**. TUNEL assay of LT. Scale bar: 50 μm **(B)**. *p < 0.05; **p < 0.01; ***p < 0.001. ^#^ *p* < 0.05; ^##^ *p* < 0.01; ^###^ *p* < 0.001. DMOG, dimethyloxalylglycine; LT, liver transplantation; PH, partial hepatectomy.

We tested the effect of DMOG pretreatment on hepatocyte apoptosis induced by IRI. Six hours after reperfusion, representative liver sections are shown in Figures 3A, B. The apoptosis event was significantly induced in the PH and LT groups, showing a higher apoptosis rate, while DMOG pretreatment significantly decreased the apoptosis rate. This result is consistent with the pathological staining.

### 3.3 DMOG pretreatment attenuated oxidative stress levels in PH and partial LT rats

**FIGURE 4:**
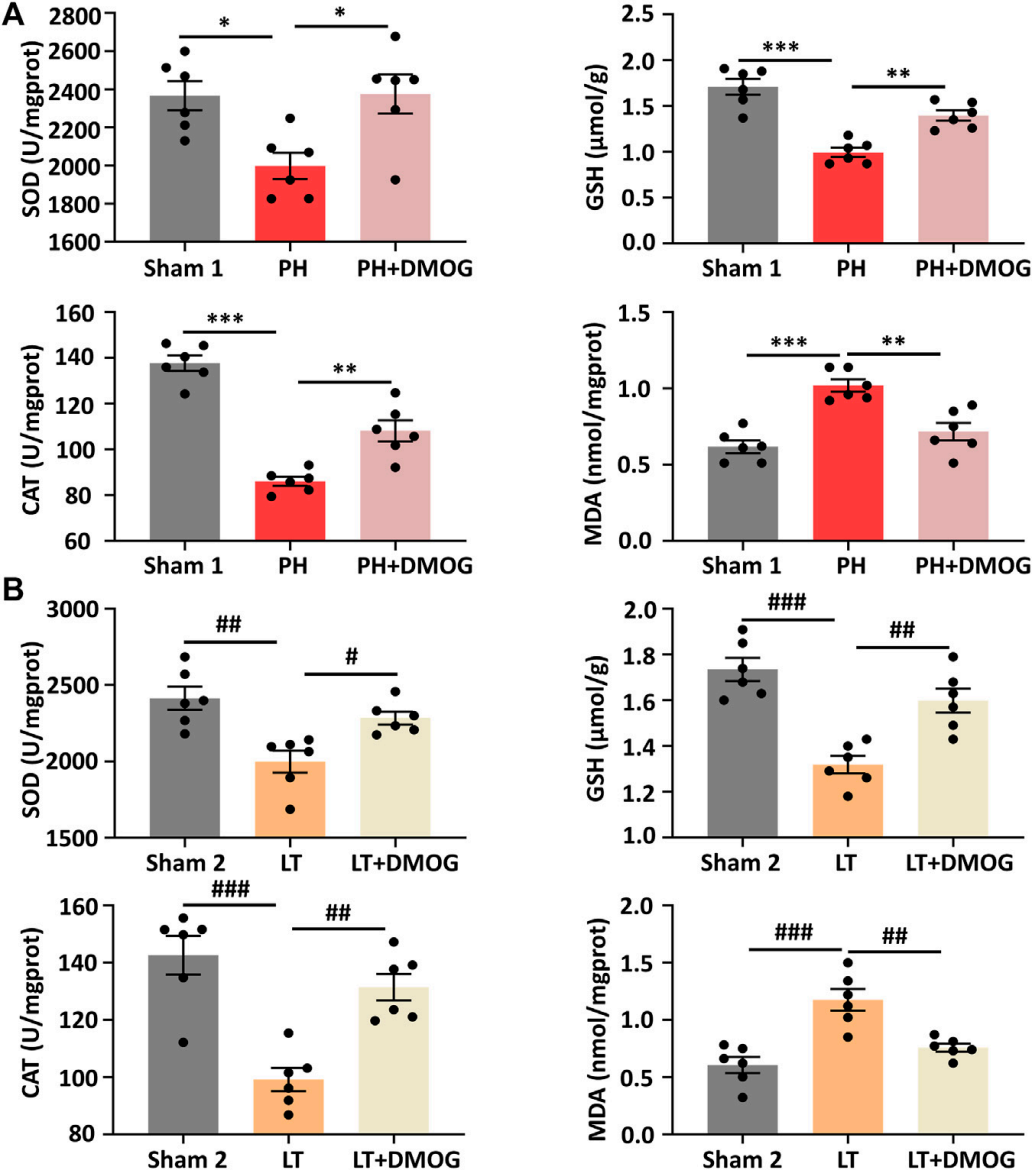
DMOG pretreatment attenuated IRI-induced oxidative stress. SOD, GSH, CAT and MDA assay of PH **(A)**. SOD, GSH, CAT and MDA assay of LT **(B)**. *p < 0.05; **p < 0.01; ***p < 0.001. ^#^ *p* < 0.05; ^##^ *p* < 0.01; ^###^ *p* < 0.001. DMOG, dimethyloxalylglycine; IRI, ischemia–reperfusion injury; LT, liver transplantation; PH, partial hepatectomy.

Oxidative stress is involved in the occurrence and development of hepatic IRI. Therefore, we studied the effects of DMOG on oxidative stress levels in PH and partial LT. PH and partial LT can induce a decrease in the antioxidant SOD, GSH and CAT and an increase in the lipid peroxidation product MDA. After DMOG treatment, the level of MDA was significantly decreased, and the levels of SOD, GSH and CAT were significantly increased (Figures 4A, B).

### 3.4 DMOG pretreatment enhanced the transcription and expression of HIF1α downstream target genes in PH and partial LT rats

**FIGURE 5:**
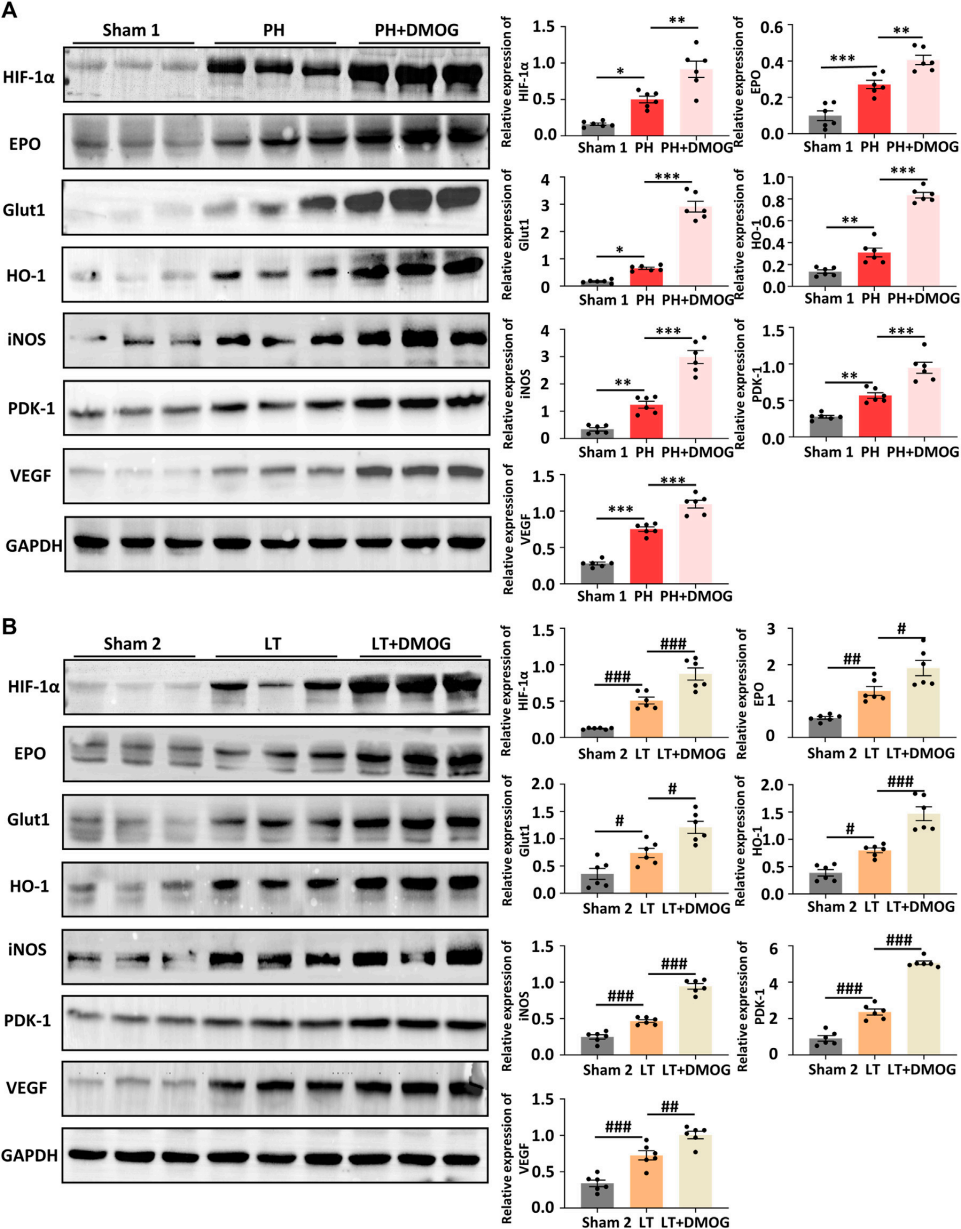
DMOG pretreatment enhanced the expression of HIF1α downstream target genes in PH and partial LT rats. Western blot analysis of HIF 1α and HIF 1 target genes in PH **(A)**. Western blot analysis of HIF 1α and HIF 1 target genes in LT **(B)**. *p < 0.05; **p < 0.01; ***p < 0.001. ^#^ *p* < 0.05; ^##^ *p* < 0.01; ^###^ *p* < 0.001. DMOG, dimethyloxalylglycine; LT, liver transplantation; PH, partial hepatectomy.

The reoxidation process is also a rapid degradation process of HIF-1α (Guo et al., 2015; Sun et al., 2018). Therefore, we set the detection time to 2 h after reperfusion. By Western blotting, we confirmed the upregulation of IRI induced by PH and partial LT on HIF1α and downstream target proteins in rats, and DMOG pretreatment further promoted this upregulation (Figures 5A, B).

**FIGURE 6:**
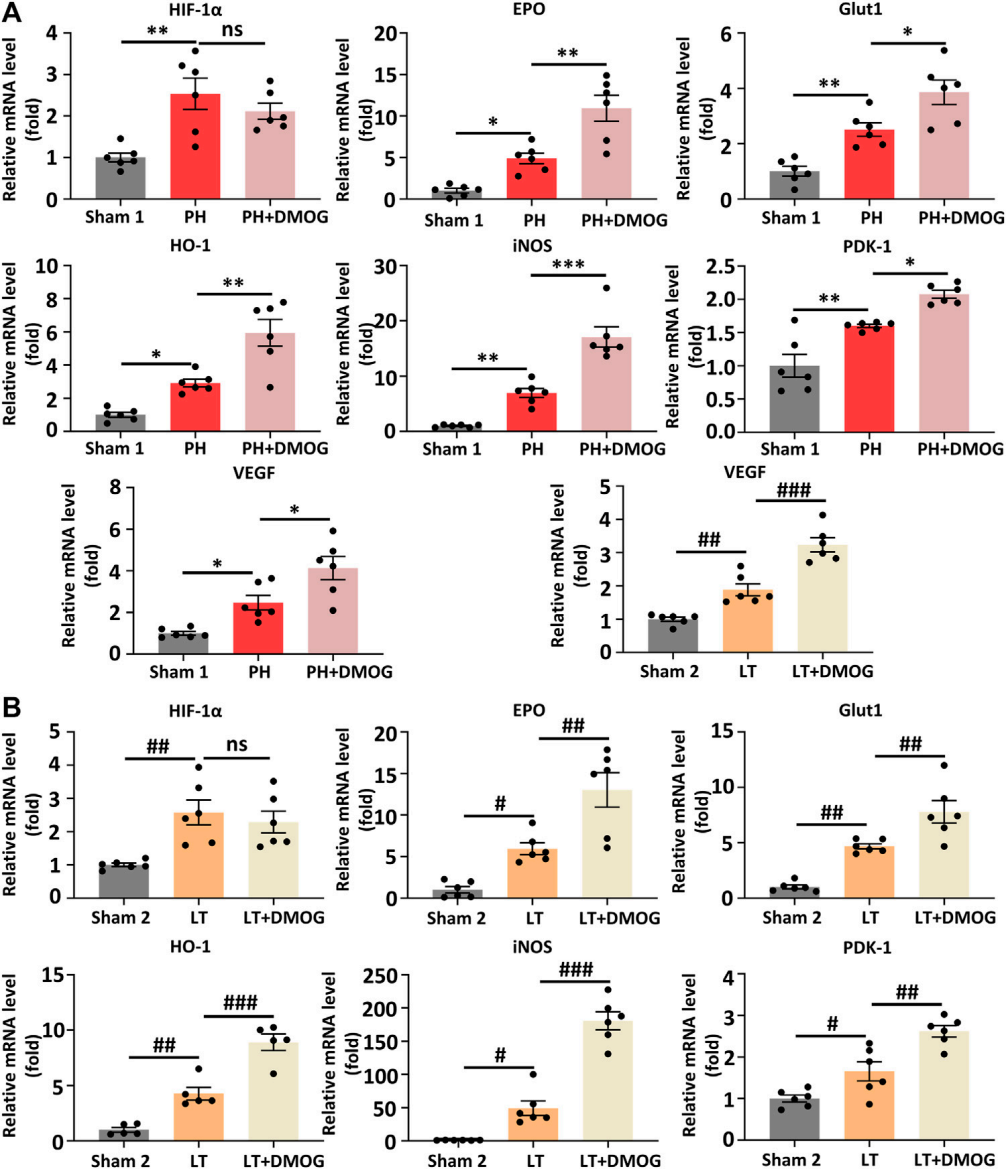
DMOG pretreatment enhanced the transcription of HIF1α downstream target genes in PH and partial LT rats. PCR analysis of HIF 1α and HIF 1 target genes in PH **(A)**. RT‒PCR analysis of HIF 1α and HIF 1 target genes in LT **(B)**. *p < 0.05; **p < 0.01; ***p < 0.001. ^#^ *p* < 0.05; ^##^ *p* < 0.01; ^###^ *p* < 0.001. DMOG, dimethyloxalylglycine; LT, liver transplantation; PH, partial hepatectomy.

To better determine the effect of DMOG on HIF-1α and HIF-1α target genes, we detected the transcription levels of HIF-1α and HIF-1α target genes by PCR. The results show that DMOG pretreatment did not significantly change HIF-1α mRNA levels, confirming that DMOG upregulates HIF-1α protein levels by inhibiting its degradation rather than promoting its expression. At the same time, mRNA levels of the HIF-1α target genes EPO, VEGF, PDK1, iNOS, GLUT1 and HO-1 were upregulated after DMOG treatment (Figures 6A, B). Together, these results demonstrate that HIF1α is stabilized and that the HIF-1 signaling pathway is activated by DMOG.

### 3.5 DMOG pretreatment improves the survival rate of partial LT recipients

**FIGURE 7:**
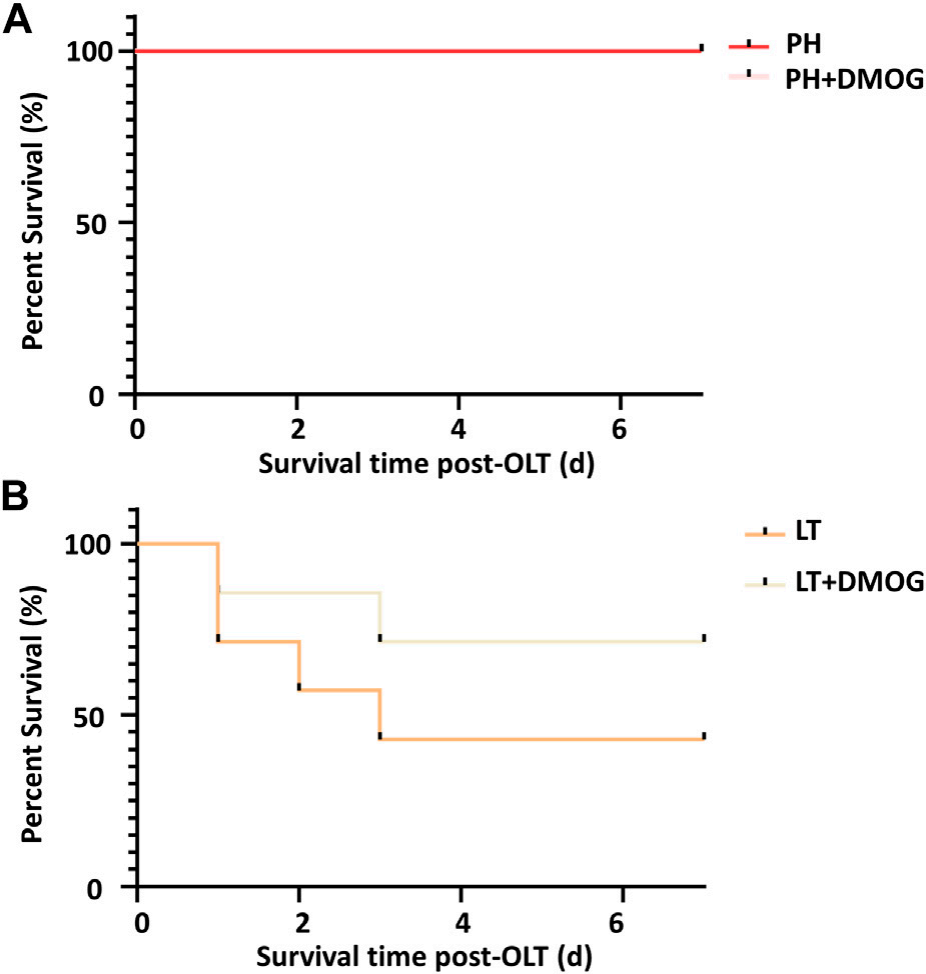
DMOG pretreatment improves the survival rate of partial LT recipients. Seven-day survival in the 70% hepatectomy **(A)** and 30% partial LT rats **(B)**. DMOG, dimethyloxalylglycine. LT, liver transplantation.

Finally, we observed the effect of DMOG on the 7-day survival of PH and partial LT rats. In the 70% hepatectomy model, both had a 100% survival rate (Figure 7A). This is consistent with previous reports that 70% hepatectomy in a standard rat model did not result in rat death (Emond et al., 1989). In the 30% LT models, DMOG improved 7-day survival from 43% to 71% compared with partial LT (Figure 7B).

## 4 Discussion

In this study, we constructed 70% hepatectomy models to simulate donors of LDLT and 30% partial LT models to simulate recipients in rats. Then, we used the HIF-1α stabilizer DMOG to pretreat the donor and found that DMOG could reduce the IRI of the donor and recipient in LDLT, which manifested as reducing liver function damage and promoting tissue recovery. In addition, we found that the HIF1 signaling pathway was activated and PH- and partial LT-induced oxidative stress levels were reduced by DMOG. Furthermore, DMOG improved the survival rate of LDLT recipients. Therefore, DMOG shows great clinical potential in the treatment of LDLT. Our study confirms that DMOG pretreatment of donors alleviates IRI in LDLT donors and recipients.

LDLT has been widely carried out around the world and has achieved almost the same effect as deceased donor LT, opening up a new method of donor liver sources for countries and regions that have not yet been legislated on brain death and can effectively alleviate the shortage of donors (Kollmann et al., 2018). However, LDLT also has to face the problem of liver IRI. IRI can lead to graft dysfunction, primary nonfunction, and even graft failure (Ito et al., 2021). The safety of the donor in LDLT cannot be ignored. Donors after circulatory death and brain death are limited by the uncertainty of acquisition time, so it is difficult to process the donor liver in advance. In the context of living organ donation, transplants are often scheduled, giving us the opportunity to intervene. “Hypoxic preconditioning” in advance provided convenience for clinical transformation. The activation of HIF 1 is considered to be an important mechanism of cellular adaptation to hypoxia. Many previous studies have reported that activation of the prerequisite HIF1 prevents ischemia-reperfusion-induced damage. Stabilizing HIF1 with the small molecule DMOG may be a novel strategy for alleviating hepatic IRI induced by liver resection and partial LT. Our study confirms that DMOG pretreatment in donors alleviates IRI in LDLT donors and recipients by stabilizing HIF1. More importantly, DMOG pretreatment improved recipient survival. At all times, higher survival is the primary goal of LT.

High levels of reactive oxygen species (ROS) are a major driver of liver cell damage. During the ischemia phase, oxidative phosphorylation and ATP synthesis are inhibited due to cell hypoxia and interruption of the mitochondrial electron transport chain, resulting in a large amount of local ROS production. During the reperfusion period, xanthine dehydrogenase is transformed into xanthine oxidase, which can bind with oxygen molecules to produce a large number of ROS, and the increased activity of nicotinamide adenine dinucleotide phosphate oxidase and the activation of Kupffer cells in the liver can also lead to a large amount of ROS production (Lemasters et al., 1995; Cadenas, 2018). Excess nonphysiological ROS can cause considerable damage to hepatocytes through lipid peroxidation, protein oxidation, mitochondrial dysfunction, and DNA damage (Sies et al., 2017). To prevent oxidative damage, organisms have evolved defense mechanisms designed to regulate ROS levels and maintain redox homeostasis. SOD, GSH and CAT are antioxidant enzymes that clear ROS in the body, while MDA is the end product of lipid peroxidation. Our results show that IRI can significantly increase the oxidation product MDA and significantly decrease the antioxidant enzymes SOD, GSH and CAT, thus destroying redox homeostasis. DMOG pretreatment restored SOD, GSH and CAT levels and decreased MDA production. In addition, after cellular damage caused by oxidative stress, damage-associated molecular patterns are released into the circulation, activating the innate immune response and further aggravating liver damage (Simpson and Oliver, 2020). Therefore, the inhibitory effect of DMOG on oxidative stress may have a deeper significance, which needs further experimental verification.

Apoptosis is an important pathological event in liver IRI and is also induced by oxidative stress (Datta et al., 2002; Conde de la Rosa et al., 2006). Ischemia itself can trigger cell apoptosis, while reperfusion can accelerate this process (Malhi et al., 2006). In this study, we detected apoptotic cells in liver slices using TUNEL. Apoptosis was observed in both the donor and recipient rat cells. After DMOG pretreatment, the apoptosis rate of cells significantly decreased, which is consistent with the results of oxidative stress. Hepatocyte apoptosis has an important impact on prognosis. A previous study retrospectively analyzed the histological evidence of liver biopsy in 35 patients after reperfusion and found that the degree of cell apoptosis increased with the increase in biochemical and pathological parameters of reperfusion injury (Kuo et al., 1998). Therefore, inhibiting cell apoptosis is one way to reduce reperfusion injury.

After DMOG treatment, the expression of HIF-1α and its downstream genes, such as EPO, VEGF, PDK-1, iNOS, Glut1 and HO-1, was enhanced. The downstream gene of HIF1α is fundamental to the liver protective effect of DMOG. Our previous studies have shown that exogenous VEGF can alleviate liver IRI (Cao et al., 2017). Downregulation of HO-1 makes the graft more susceptible to IRI, and targeting HO-1 has also been shown to alleviate IRI (Amersi et al., 1999; Nakamura et al., 2017). The upregulation of EPO, PDK1 and GLUT1 all has an inhibitory effect on IRI (Greif et al., 2010; Schmeding et al., 2010; Iwata et al., 2014; Ong et al., 2014; Wang et al., 2020). The role of iNOS in hepatic IRI has been controversial, and NO induced by iNOS may have protective or harmful effects, which needs to be further explored (Zhang et al., 2017). However, overall, stabilizing HIF1α alleviated IRI in PH and partial LT.

There are still some shortcomings in this study. First, the dose selected is based on previous studies, and a dose‒response curve for liver protection needs to be determined to determine the optimal dose and timing of DMOG administration and whether multiple dosing before ischemia is more effective. Second, DMOG promotes the transcription and expression of downstream HIF1 target genes; however, the size and relative importance of these genes’ effects on liver protection are not well understood. Hopefully, future research will address these problems.

## 5 Conclusion

To the best of our knowledge, this is the first animal study to focus on donors and recipients of LDLT at the same time and the first to examine the effects of DMOG on LDLT. This study demonstrated the beneficial effects of DMOG pretreatment on donors and recipients in LDLT through 70% liver resection and 30% partial LT models, which was related to the stabilization of HIF-1 and activation of the HIF1 signaling pathway. This study provides evidence for further clinical transformation of DMOG.

## Data Availability

The original contributions presented in the study are included in the article/Supplementary Material, further inquiries can be directed to the corresponding author.
